# Supplementary material for: The methyl phosphate capping enzyme Bmc1/Bin3 is a stable component of the fission yeast telomerase holoenzyme
Source: Nat Commun. 2022 Mar 11;13:1277. doi: 10.1038/s41467-022-28985-3 (PMC8917221; doi:10.1038/s41467-022-28985-3)
Supplement: Supplementary file 1 — Supplementary Information [file 41467_2022_28985_MOESM1_ESM.pdf]

Supplementary Information for "The methyl phosphate capping enzyme Bmc1/Bin3 is a stable component of the fission yeast telomerase holoenzyme"

Jennifer Porat[1], Moaine El Baidouri[2,3], Jorg Grigull[4], Jean-Marc Deragon[2,3,5], & Mark A. Bayfield[1*]

1. Department of Biology, York University, Toronto, Canada

2. LGDP-UMR5096, Université de Perpignan Via Domitia, Perpignan, France

3. CNRS LGDP-UMR5096, Perpignan, France

4. Department of Mathematics and Statistics, York University, Toronto, Canada

5. Institut Universitaire de France, Paris, France

*Correspondence: bayfield@yorku.ca (M.A.B.)

Supplementary figure 1: Bmc1 is the *S. pombe* methyl phosphate capping enzyme (MePCE) homolog

Supplementary figure 2: Reciprocal co-immunoprecipitations validate an interaction between Bmc1 and Pof8

Supplementary figure 3: Glycerol gradient sedimentation of Bmc1, Pof8, and TER1 in a wild type and *pof8Δ* strain

Supplementary figure 4: Bmc1 overexpression fails to rescue *pof8Δ*-dependent telomere defects

Supplementary figure 5: Validation of a Bmc1 knockout strain confirms its viability

Supplementary figure 6: Bmc1 and Pof8 are not strictly required for telomere maintenance

Supplementary figure 7: Phylogenic distribution of Bmc1/Bin3 and Pof8 in fungi

Supplementary table 1: Gene ontology analysis (cellular component) of the top 50 Bmc1 protein interactors

Supplementary table 2: Distribution of Bmc1 and Pof8 in basal eukaryotic lineages. Presence of a Bmc1- or LARP7-family member= Y, absence= N.

Supplementary table 3: Primers used to generate tagged strains

Supplementary table 4: *S. pombe* strains used in this study

Supplementary table 5: Probe sequences

S1

A

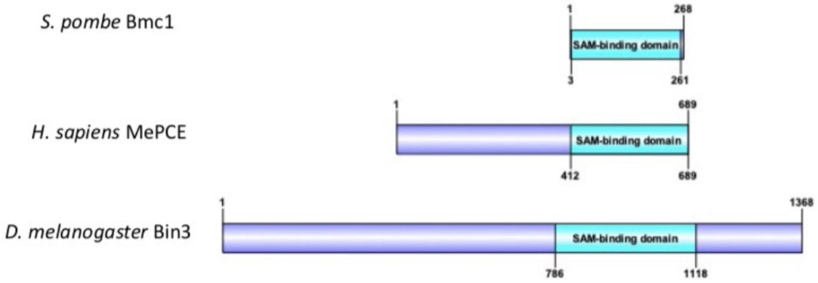

B

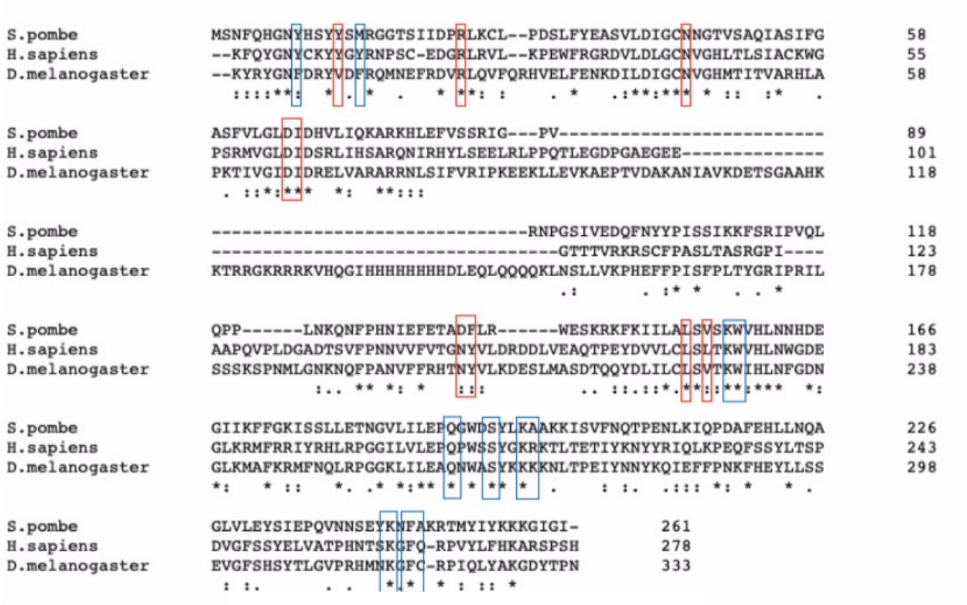

C

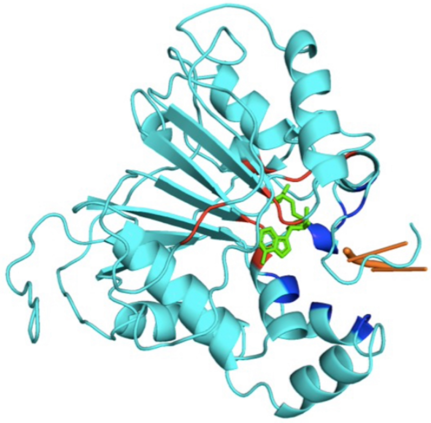

Supplementary figure 1: Bmc1 is the S. pombe methyl phosphate capping enzyme (MePCE) homolog
A) Schematic of the domain architecture of Bmc1 homologs from S. pombe, H. sapiens, and D. melanogaster.
B) Clustal alignment of the methyltransferase/SAM-binding domains of S. pombe Bmc1, H. sapiens MePCE, and D. melanogaster Bin3. Predicted SAM-binding residues are indicated in red and nucleotide-binding residues are in blue.
C) Phyre268 structure prediction of S. pombe Bmc1 bound to a 5' terminal guanosine (orange) and SAH (green). Predicted SAM-binding residues are in red and nucleotide-binding residues are in blue.

S2

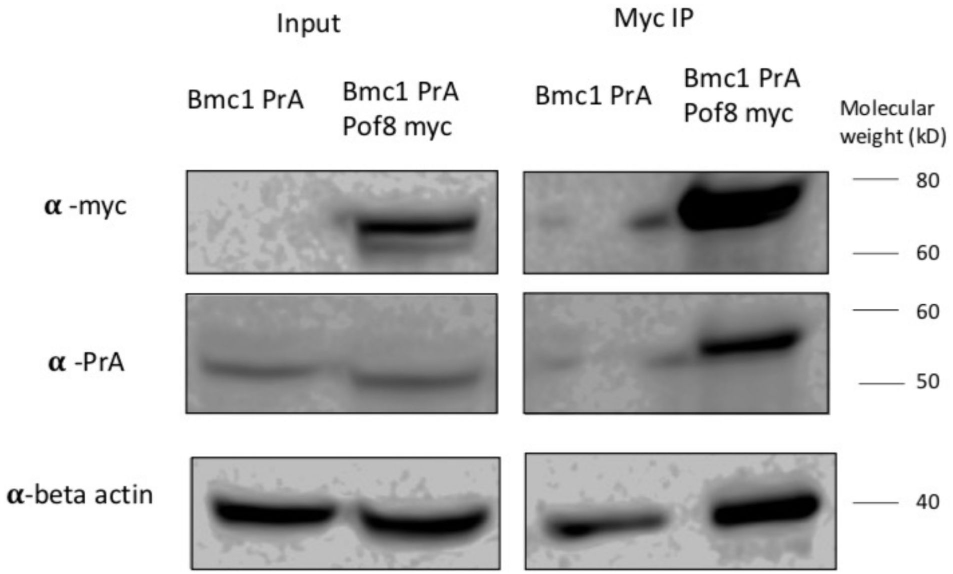

Supplementary figure 2: Reciprocal co-immunoprecipitations validate an interaction between Bmc1 and Pof8

The interaction between Bmc1 and Pof8 was confirmed by α-myc co-immunoprecipitation. Source data are provided as a Source Data file.

S3

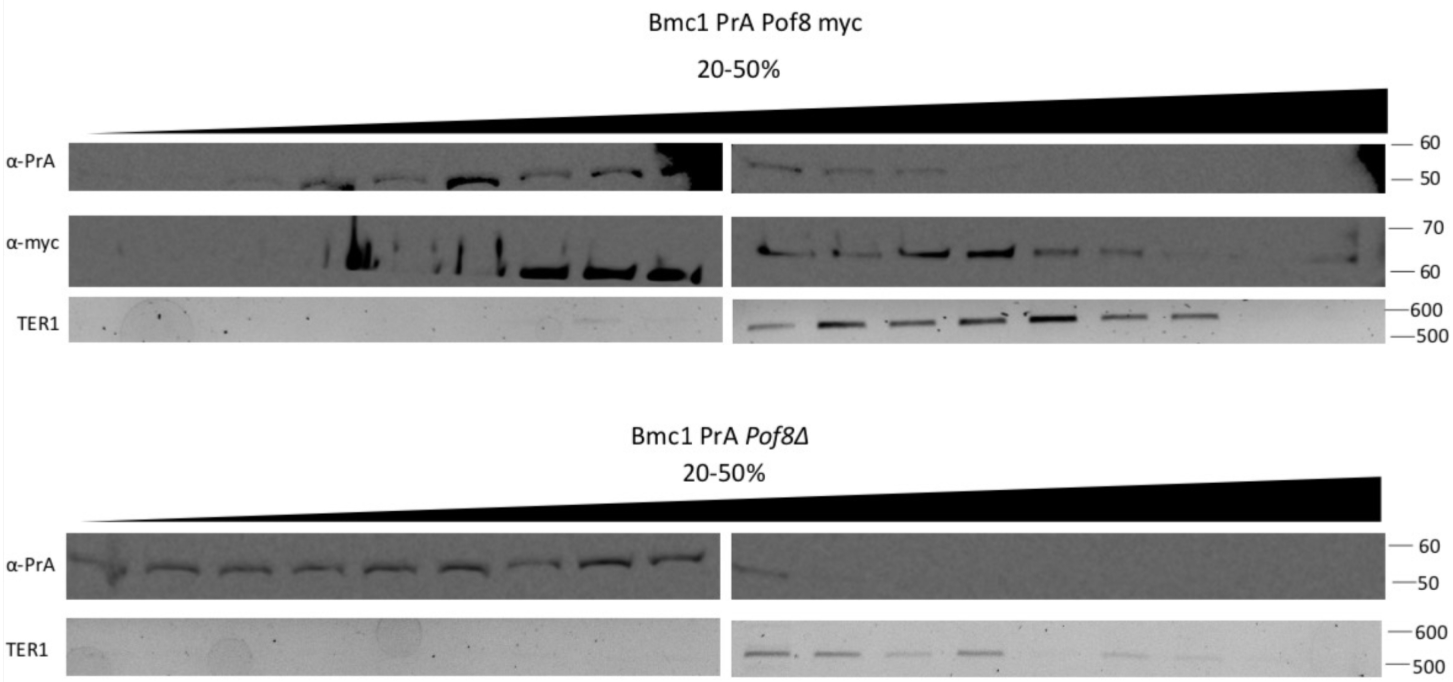

Supplementary figure 3: Glycerol gradient sedimentation of Bmc1, Pof8, and TER1 in a wild type and pof8Δ strain
Western blot and semi-quantitative RT-PCR of Bmc1 PrA, Pof8 myc, and TER1 in gradient fractions. Source data are provided as a Source Data file.

S4

A

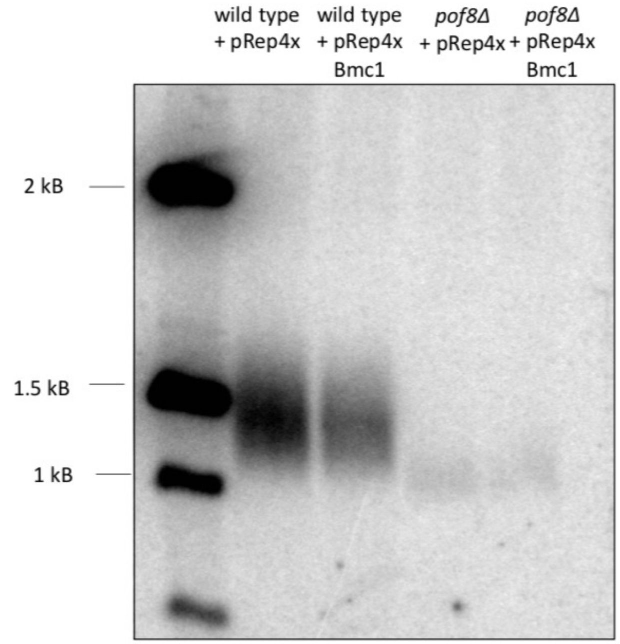

B

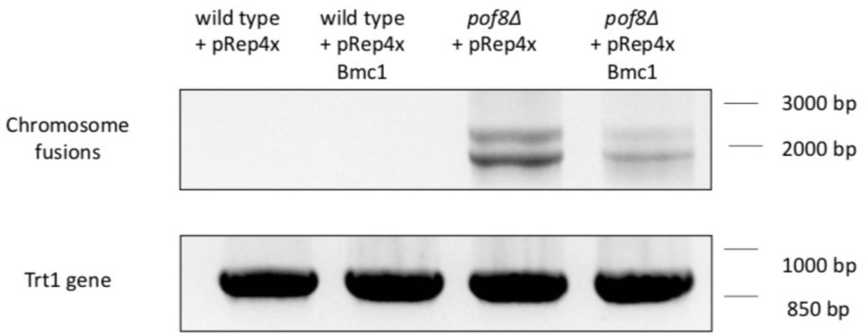

Supplementary figure 4: Bmc1 overexpression fails to rescue pof8Δ-dependent telomere defects
A) Southern blot comparing telomere length from wild type and pof8Δ strains following 2 restreaks on minimal media (one re-streak= 20-25 generations) overexpressing an empty vector (pRep4x) or Bmc1. Genomic DNA was digested with EcoRI.
B) Chromosome fusion PCR (top). The Trt1 gene was amplified from genomic DNA as a loading control (bottom). Template DNA from the same genomic DNA as (A). Source data are provided as a Source Data file.

S5

A

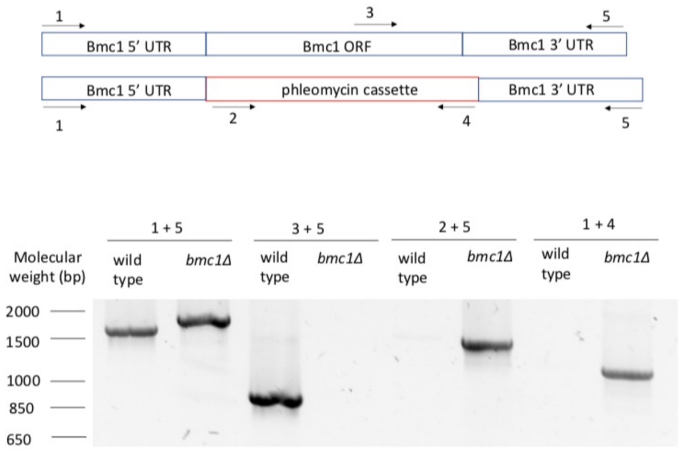

B

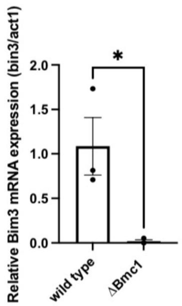

C

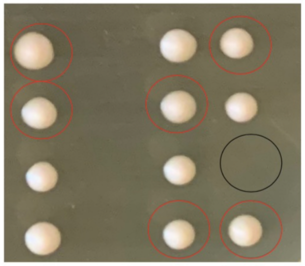

D

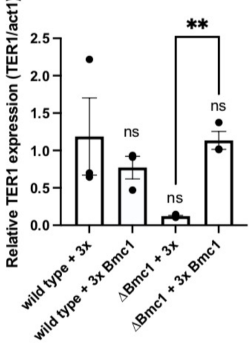

Supplementary figure 5: Validation of a Bmc1 knockout strain confirms its viability

A) Schematic of Bmc1 knockout (top) and PCR validation of the resultant strain using the indicated primers.
B) qRT-PCR of bmc1 mRNA normalized to act1 mRNA for wild type and bmc1Δ strains (mean ± standard error, unpaired t test * at $p < 0.05$, ** at $p < 0.01$, *** at $p < 0.001$, and **** at $p<0.0001$) (n=3 biological replicates). P-values over each bar represent results of a two-tailed unpaired t-test with a wild type strain.
C) Backcrossing of bmc1Δ and a wild type strain, followed by tetrad dissection, confirms viability of the bmc1Δ strain on rich media. Tetrads from the same spore are arranged in columns. Resulting haploid colonies possessing phleomycin resistance are circled in red and colonies that didn't grow after tetrad dissection are circled in black.
D) qRT-PCR of TER1 normalized to act1 mRNA for wild type and bmc1Δ strains transformed with an empty vector (3x) or Bmc1 (mean ± standard error, two-tailed unpaired t test * at $p < 0.05$, ** at $p < 0.01$, *** at $p < 0.001$, and **** at $p<0.0001$) (n=3 biological replicates). P-values over each bar represent results of a two-tailed unpaired t-test with a wild type strain transformed with an empty vector (wild type + pRep3x). Source data are provided as a Source Data file.

S6

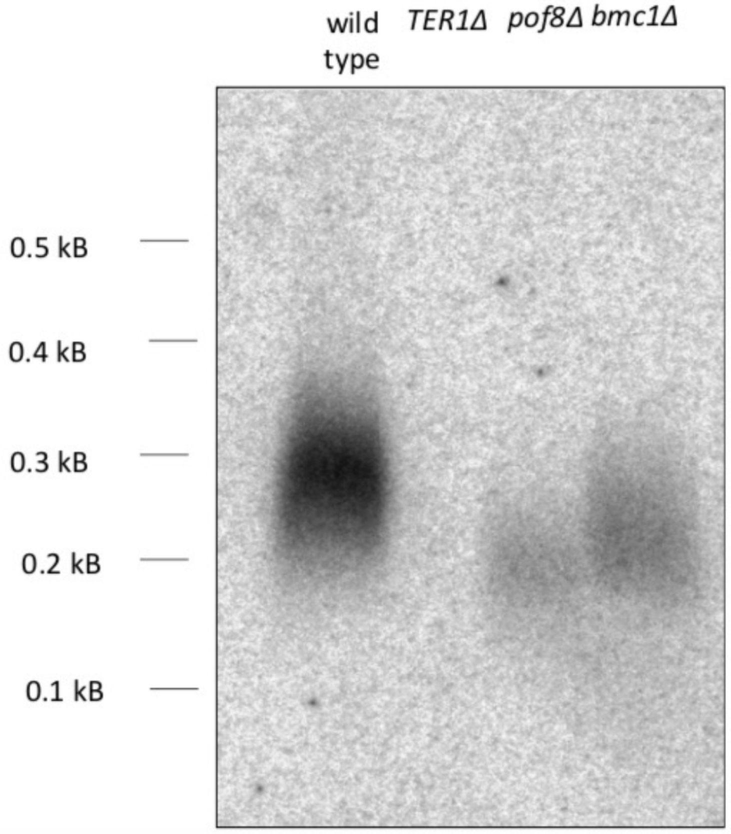

Supplementary figure 6: Bmc1 and Pof8 are not strictly required for telomere maintenance
Southern blot comparing ApaI-digested genomic DNA isolated after 5 restreaks on rich media. A lack of signal with a telomeric probe represents survivors that have lost telomeric DNA and circularized their chromosomes (TER1Δ). Source data are provided as a Source Data file.

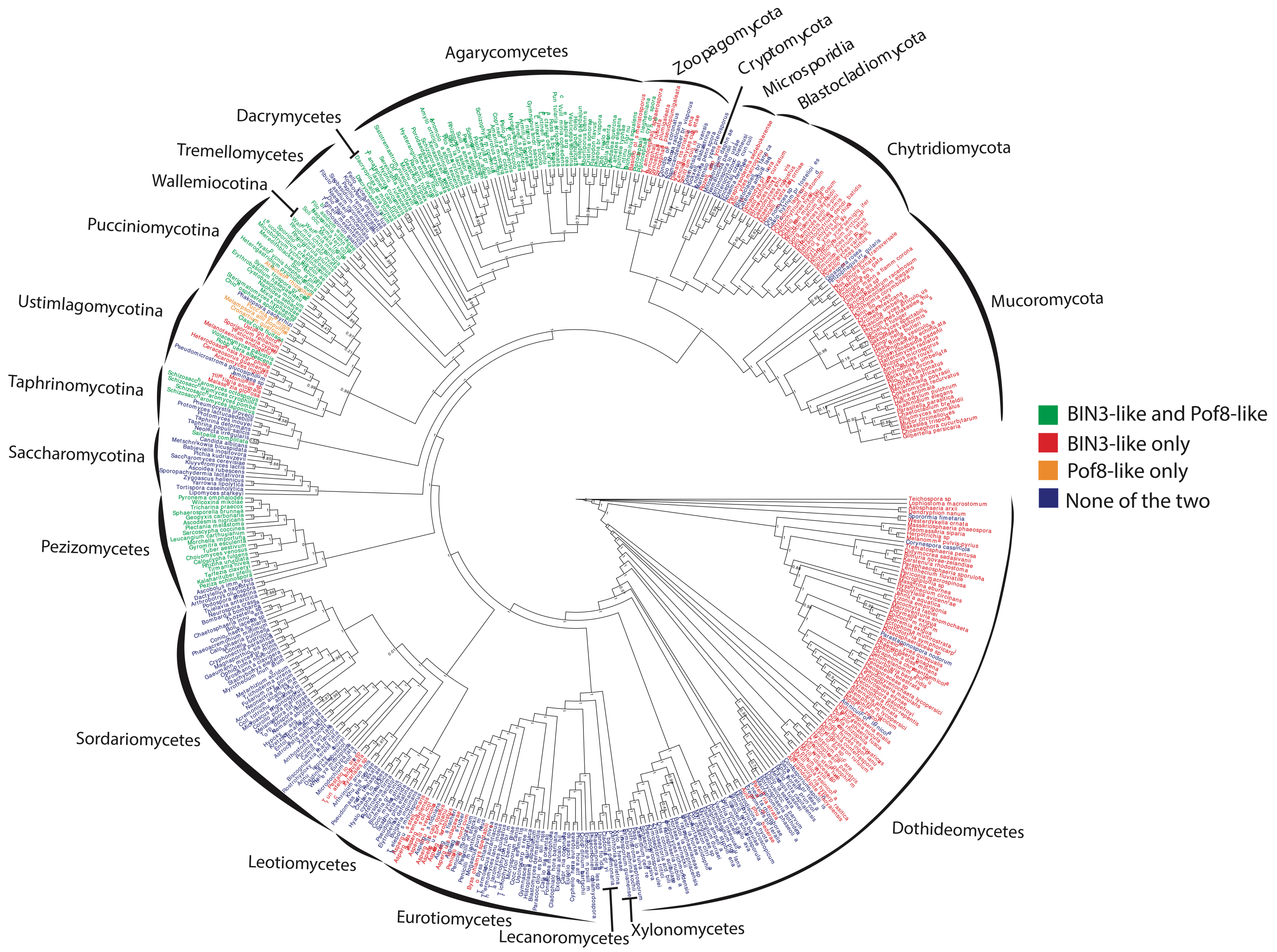

Supplementary figure 7: Phylogenic distribution of Bmc1/Bin3 and Pof8 in fungi
Complete consensus cladogram describing the phylogenic relationships of 472 species representative of fungi phylum and classes and highlighting (using a color code) the distribution of Bin3 and Pof8 in these species. Only posterior probabilities inferior to 1 are shown.

Supplementary table 1: Gene ontology analysis (cellular component) of the top 50 Bmc1 protein interactors

GO cellular component	Fold enrichment	Raw P value	False discovery rate
Lsm2-8 complex	42.34	8.86E-06	1.23E-03
Telomere holoenzyme complex	41.16	6.60E-07	2.44E-04
U6 snRNP	37.04	1.32E-05	1.63E-03
Spliceosomal tri-snRNP complex	15.88	4.49E-06	9.97E-04
U4/U6 x U5 tri-snRNP complex	15.88	4.49E-06	8.31E-04
Catalytic step 2 spliceosome	10.01	2.10E-04	1.46E-02
Spliceosomal snRNP complex	8.50	2.78E-05	2.80E-03
Small nuclear ribonucleoprotein complex	8.10	3.69E-05	3.42E-03
Sm-like protein family complex	7.98	4.05E-05	3.46E-03
Spliceosomal complex	6.12	7.25E-06	1.15E-03

Supplementary table 2: Distribution of Bmc1 and Pof8 in basal eukaryotic lineages. Presence of a Bmc1- or LARP7-family member= Y, absence= N.

Species	Classification	BIN3	LARP7
Fragilariopsis cylindrus	Stramenopiles, Bacillariophyta	N	N
Pseudo-nitzschia multiseries	Stramenopiles, Bacillariophyta	N	N
Thalassiosira oceanica	Stramenopiles, Bacillariophyta	N	N
Thalassiosira pseudonana	Stramenopiles, Bacillariophyta	N	N
Ochromonadaceae sp	Stramenopiles, Chrysophyta	Y	N
Nannochloropsis gaditana	Stramenopiles, Eustigmatophyceae	Y	N
Nannochloropsis oceanica	Stramenopiles, Eustigmatophyceae	Y	N
Aplanochytrium kerguelense	Stramenopiles, Labyrinthulomycetes	N	N
Aurantiochytrium limacinum	Stramenopiles, Labyrinthulomycetes	N	N
Schizochytrium aggregatum	Stramenopiles, Labyrinthulomycetes	N	N
Achlya hypogyna	Stramenopiles, Oomycota	Y	Y
Aphanomyces euteiches	Stramenopiles, Oomycota	Y	Y
Aphanomyces astaci	Stramenopiles, Oomycota	Y	Y
Phytophthora cactorum	Stramenopiles, Oomycota	y	y
Phytophthora capsici	Stramenopiles, Oomycota	y	y
Phytophthora cinnamomi	Stramenopiles, Oomycota	y	y
Phytophthora infestans	Stramenopiles, Oomycota	y	y
Phytophthora sojae	Stramenopiles, Oomycota	y	y
Plasmopara halstedii	Stramenopiles, Oomycota	y	y
Pythium brassicum	Stramenopiles, Oomycota	Y	Y
Saprolegnia parasitica	Stramenopiles, Oomycota	Y	Y
Thraustotheca clavata	Stramenopiles, Oomycota	Y	Y
Blastocystis hominis	Stramenopiles, Opalozoa	N	N
Pelagophyceae sp.	Stramenopiles, Pelagophyceae	Y	Y
Ectocarpus siliculosus	Stramenopiles, Phaeophyceae	Y	Y
Tetrahymena thermophila	Alveolates, Ciliophora	Y	Y
Paramecium tetraurelia	Alveolates, Ciliophora	Y	Y
Pseudocohnilembus persalinus	Alveolates, Ciliophora	Y	N
Oxytricha trifallax	Alveolates, Ciliophora	Y	Y
Symbiodinium microadriaticum	Alveolates, Dinophyta	Y	N
Stylonychia lemnae	Alveolates, Ciliophora	Y	Y
Stentor coeruleus	Alveolates, Ciliophora	Y	N
Perkinsus marinus	Alveolates, Perkinsea	Y	N

Vitrella brassicaformis	Alveolates, Chromorida	Y	N
Gregarina niphandrodes	Alveolates, Apicomplexa	N	N
Cryptosporidium muris	Alveolates, Apicomplexa	N	N
Eimeria acervulina	Alveolates, Apicomplexa	Y	N
Toxoplama gondii	Alveolates, Apicomplexa	Y	N
Neospora caninum	Alveolates, Apicomplexa	Y	N
Cyclospora cayetanensis	Alveolates, Apicomplexa	N	N
Cystoisospora suis	Alveolates, Apicomplexa	Y	N
Plasmodiophora brassicae	Rhizaria, Endomixa	Y	N
Trypanosoma brucei	Excavates, Euglenozoa	N	N
Naegleria gruberi	Excavates, Heterolobosea	Y	N
Naegleria fowleri	Excavates, Heterolobosea	Y	N
Giardia lamblia	Excavates, Fornicata	N	N
Giardia intestinalis	Excavates, Fornicata	N	N
Trichomonas vaginalis	Excavates, Parabasalia	N	N
Emiliania huxleyi	Haptophyta, Isochrysidales	Y	Y
Chrysochromulina tobinii	Haptophyta, Prymnesiales	N	N
Pavlovalas sp	Haptophyta, Pavlovales	Y	N
Phaeocystis globosa	Haptophyta, Phaeocystales	Y	N

Supplementary table 3: Primers used to generate tagged strains

Primer	Sequence
Bmc1 PrA For (tag integration)	5'CATTTGCTTAATCAAGCAGGACTAGTGCTTGAATACAGTATCGAACCTCA AGTAAATAACTCTGAGTACAAGAATTTTGCCAAACGAACAATGTATAAAAA AAAAGGAATTGGAATCATAAAACTATTAACTATTAACTTCTACTCGTACGCT GCAGGTCGAC 3'
Bmc1 PrA Rev (tag integration)	5'CTACTTCATTCCGCTTTTCTTTCTTTCCTCATATGTTGAATTAGAGAATTTAAT AAAAAATGAGACACGTCCTTTTGGGACATTATGCTTTATAGTTCCAGTCATTGT CTATTCTTAACGTTTTACTAGACAAATCGATGAATTCGATGAATTCGAGCTCG 3'
MX6 For (antibiotic marker swap)	5' GACATGGAGGCCCAGAATAC 3'
MX6 Rev (antibiotic marker swap)	5' TGGATGCGCGCGTTAGTATC 3'
5' Bmc1 SalI For (Bmc1 KO)	5' GCGCGTCGACCTTACAACAACAAATTCTAACCTTAGCTACTTTAGGAGG 3'
5' Bmc1 BglII Rev (Bmc1 KO)	5'GCGCAGATCTCCCAAGTCGAGGAGGTTTTTCAAATCACCCCTATTTTTTATAAAAG 3'
3' Bmc1 ClaI For (Bmc1 KO)	5' GCGCATCGATTTGTCTAGTAAAACGTTAAGAATAGACAATGACTGGAACTATAAAG 3'
3' Bmc1 SacII Rev (Bmc1 KO)	5' GCGCCCGCGGGGGTGATGTTGGCGAGTATAACCAATGTCAAACTCAATTATTTC 3'

Supplementary table 4: *S. pombe* strains used in this study

Figure	Strain	Description	Full genotype	Source
1, Data S1	y12088	wild type	*h+ ura4-D18 leu1-32*	Lab stock
	yJP001	*bmc1-PrA*	*h- ura4-D18 bmc1^{+}::bmc1-PrA-kanMX6*	This study
2	FY6843	wild type	*h- ura4-D18*	National BioResource Project
	yJP001	*bmc1-PrA*	*h- ura4-D18 bmc1^{+}::bmc1-PrA-kanMX6*	This study
	TN7706	*trt1-myc*	*h- leu1-32 ura4-D18 his3-D1 trt1^{+}::G8-13myc-natMX6*	Mennie *et al.*, 2018
	AM16932	*pof8-myc*	*h- leu1-32 ura4-D18 his3-D1 pof8^{+}::13myc-kanMX6*	Mennie *et al.*, 2018
	AM17121	*lsm3-myc*	*h- leu1-32 ura4-D18 his3-D1 lsm3^{+}::13myc-kanMX6*	Mennie *et al.*, 2018
3a, Data S2, Table S1	FY6843	wild type	*h- ura4-D18*	National BioResource Project
	yJP001	*bmc1-PrA*	*h- ura4-D18 bmc1^{+}::bmc1-PrA-kanMX6*	This study
3b, S2	yJP001	*bmc1-PrA*	*h- ura4-D18 bmc1^{+}::bmc1-PrA-kanMX6*	This study
	yJP001a	*bmc1-PrA*	*h+ ura4-D18 bmc1^{+}::bmc1-PrA-kanMX6*	This study
	yJP001b	*bmc1-PrA*	*h+ ura4-D18 bmc1^{+}::bmc1-PrA-bleMX6*	This study
	AM16932	*pof8-myc*	*h- leu1-32 ura4-D18 his3-D1 pof8^{+}::13myc-kanMX6*	Mennie *et al.*, 2018
	yJP012	*bmc1-PrA pof8-myc*	*h- leu1-32 ura4-D18 his3-D1 bmc1^{+}::bmc1-PrA-bleMX6 pof8^{+}::13myc-kanMX6*	This study
	TN17480	*ter1Δ*	*Leu1-32::[ter1+ leu1+] ura4-D18 his3-D1 ter1Δ25-1135:ura4+:tk+*	Mennie *et al.*, 2018
	yJP025	*bmc1-PrA pof8-myc ter1Δ*	*h- ura4-D18 his3-D1 bmc1^{+}::bmc1-PrA-bleMX6 pof8^{+}::13myc-kanMX6 ter1Δ25-1135:ura4+:tk+*	This study

3c	yJP001	*bmc1-PrA*	*h- ura4-D18 bmc1^{+}::bmc1-PrA-kanMX6*	This study
	yJP001a	*bmc1-PrA*	*h+ ura4-D18 bmc1^{+}::bmc1-PrA-kanMX6*	This study
	yJP001b	*bmc1-PrA*	*h+ ura4-D18 bmc1^{+}::bmc1-PrA-bleMX6*	This study
	TN7706	*trt1-myc*	*h- leu1-32 ura4-D18 his3-D1 trt1^{+}::G8-13myc-natMX6*	Mennie *et al.,* 2018
	yJP013	*bmc1-PrA trt1-myc*	*h- leu1-32 ura4-D18 his3-D1 bmc1^{+}::bmc1-PrA-bleMX6 trt1^{+}::G8-13myc-natMX6*	This study
	TN17480	*ter1Δ*	*Leu1-32::[ter1+ leu1+] ura4-D18 his3-D1 ter1Δ25-1135:ura4+:tk+*	Mennie *et al.,* 2018
	yJP021	*bmc1-PrA trt1-myc ter1Δ*	*h- leu1-32 ura4-D18 his3-D1 bmc1^{+}::bmc1-PrA-bleMX6 trt1^{+}::G8-13myc-kanMX6 ter1Δ25-1135:ura4+:tk+*	This study
4a,b	FY6843	wild type	*h- ura4-D18*	National BioResource Project
	yJP001	*bmc1-PrA*	*h- ura4-D18 bmc1^{+}::bmc1-PrA-kanMX6*	This study
	yJP001a	*bmc1-PrA*	*h+ ura4-D18 bmc1^{+}::bmc1-PrA-kanMX6*	This study
	yJP001b	*bmc1-PrA*	*h+ ura4-D18 bmc1^{+}::bmc1-PrA-bleMX6*	This study
	TN12118	*pof8Δ*	*h- leu1-32 ura4-D18 his3-D1 pof8Δ::kanMX6*	Mennie *et al.,* 2018
	TN12118a	*pof8Δ*	*h- leu1-32 ura4-D18 his3-D1 pof8Δ::natMX6*	This study
	yJP011	*bmc1-PrA pof8Δ*	*h- leu1-32 ura4-D18 his3-D1 bmc1^{+}::bmc1-PrA-bleMX6 pof8Δ::natMX6*	This study
4c	FY6843	wild type	*h- ura4-D18*	National BioResource Project
	yJP001	*bmc1-PrA*	*h- ura4-D18 bmc1^{+}::bmc1-PrA-kanMX6*	This study
	TN7706	*trt1-myc*	*h- leu1-32 ura4-D18 his3-D1 trt1^{+}::G8-13myc-natMX6*	Mennie *et al.,* 2018

	AM16932	*pof8-myc*	*h- leu1-32 ura4-D18 his3-D1 pof8⁺::13myc-kanMX6*	Mennie *et al.,* 2018
	AM17121	*lsm3-myc*	*h- leu1-32 ura4-D18 his3-D1 lsm3⁺::13myc-kanMX6*	Mennie *et al.,* 2018
	yJP001	*bmc1-PrA*	*h- ura4-D18 bmc1⁺::bmc1-PrA-kanMX6*	This study
	yJP001a	*bmc1-PrA*	*h+ ura4-D18 bmc1⁺::bmc1-PrA-kanMX6*	This study
	yJP001b	*bmc1-PrA*	*h+ ura4-D18 bmc1⁺::bmc1-PrA-bleMX6*	This study
	TN12118	*pof8Δ*	*h- leu1-32 ura4-D18 his3-D1 pof8Δ::kanMX6*	Mennie *et al.,* 2018
	TN12118a	*pof8Δ*	*h- leu1-32 ura4-D18 his3-D1 pof8Δ::natMX6*	This study
	yJP011	*bmc1-PrA pof8Δ*	*h- leu1-32 ura4-D18 his3-D1 bmc1⁺::bmc1-PrA-bleMX6 pof8Δ::natMX6*	This study
S3	yJP012	*bmc1-PrA pof8-myc*	*h- leu1-32 ura4-D18 his3-D1 bmc1⁺::bmc1-PrA-bleMX6 pof8⁺::13myc-kanMX6*	This study
	yJP011	*bmc1-PrA pof8Δ*	*h- leu1-32 ura4-D18 his3-D1 bmc1⁺::bmc1-PrA-bleMX6 pof8Δ::natMX6*	This study
S4	y12088	wild type	*h+ ura4-D18 leu1-32*	Lab stock
	TN12118	*pof8Δ*	*h- leu1-32 ura4-D18 his3-D1 pof8Δ::kanMX6*	Mennie *et al.,* 2018
S5	yAS99	wild type	*h- leu1-32 ura4-D18 ade6-704*	Intine et al., 2000, Mol Cell
	yJP022	*bmc1Δ*	*h+ ura4-D18 leu1-32 bmc1Δ::bleMX6*	This study
5a, b	y12088	wild type	*h+ ura4-D18 leu1-32*	Lab stock
	yJP022	*bmc1Δ*	*h+ ura4-D18 leu1-32 bmc1Δ::bleMX6*	This study
	TN12118	*pof8Δ*	*h- leu1-32 ura4-D18 his3-D1 pof8Δ::kanMX6*	Mennie *et al.,* 2018
	FBY195	*rrp6Δ*	*h- ade6-704 ura4- leu1-32 rrp6Δ::kanMX6*	Huang *et al.,* 2006, NSMB
	yJP024	*pof8Δ rrp6Δ*	*h? ade6-704 ura4- leu1-32 rrp6Δ::kanMX6 pof8ΔnatMX6*	This study
	yJP026	*bmc1Δ rrp6Δ*	*h- ade6-704 ura4- leu1-32 rrp6Δ::kanMX6 bmc1ΔbleMX6*	This study

5c,d,e,f	AM16932	*pof8-myc*	*h- leu1-32 ura4-D18 his3-D1 pof8*$^{+}$*::13myc-kanMX6*	Mennie *et al.,* 2018
	yJP023	*pof8-myc bmc1Δ*	*h+ ura4-D18 leu1-32 his3-D1 pof8*$^{+}$*::13myc-kanMX6 bmc1Δ::bleMX6*	This study
S6	y12088	wild type	*h+ ura4-D18 leu1-32*	Lab stock
	AM17307	*ter1Δ*	*h+ leu1-32 ura4-D18 his3-D1 ter1Δ25-1135:ura4+:tk+*	Mennie *et al.,* 2018
	TN12118	*pof8Δ*	*h- leu1-32 ura4-D18 his3-D1 pof8Δ::kanMX6*	Mennie *et al.,* 2018
	yJP022	*bmc1Δ*	*h+ ura4-D18 leu1-32 bmc1Δ::bleMX6*	This study

**Figure**	**Plasmid**
5, S5	pFA6a bleMX6
S4	pRep4x (Amp)
	pRep4x-Bmc1 (Amp)
S5	pRep3x (Amp)
	pRep3x HA-Bmc1 (Amp)

Supplementary table 5: Probe sequences

Figure	Probe	Sequence
1c, 1d, 2b, 3c, 2d, 4a, 4c	U6	5' GTGTCATCCTTGTGCAGGGGCCAT 3'
1c, 4c	U6 intron	5' CAACGAGTTAGTATGACTCGAACCTTGG 3'
1d	Ter1	5' CCCGTTCCTTTTAACATTTTGCTCAGACCAAGTG 5'
1d, 2c	U5	5' CTGGTAAAAGGCAAGAAACAGATACG 3'
2a	Ter1 cRACE For	5' TCTTGAGCGCGTTTTAGGTTTTTTTCACTT 3'
	Ter1 cRACE Rev	5' AACAACGGACGAGCTACACTCAAAATATTA 3'
2b, 4a	Ter1 3' RNase H	5' GGGTAGTCAACTATTTTACTAGCG 3'
2b, 2d, 4a	Ter1 5' RNase H	5' TTTCAAATCAATCACCAAGCCC 3'
2b, 4a	Ter1 3' RNase H northern	5' GCTCAGACCAAGTGAAAAAAACC 3'
2b, 2d, 4a	Ter1 5' RNase H northern	5' TCCTTGTCAACCTTCTAAGCATG 3'
2c	tRNA Thr^{CGU}	5' TGCTCCGGCAGTGACTCGAACA 3'
4d, 5d	Telomerase assay primer	5' GTTACGGTTACAGGTTACG 3'
S5	5' Bmc1 mRNA qPCR	5' GAGGAACCTCAATTATCGATCCAAGACTTAAATGTTTGC 3'
	3' Bmc1 mRNA qPCR	5' TCGAGCTTTTTGGATGAGAACATGATCGATATCCAATCC 3'
5a	5' act1 qPCR	5' TGCTCCTCCTGAGCGTAAATA 3'
	3' act1 qPCR	5' CCGCTCTCATCATACTCTTGC 3'
1c, 4a, 5a	5' Ter1 qPCR	5' CATTTAAGTGCTTGTCAGATCACAACG 3'
	3' Ter1 qPCR	5' ACAACGGACGAGCTACACTC 3'
5a	U6 5' qPCR	5' CGGATCACTTTGGTCAAATTG 3'
	U6 3' qPCR	CTCTCAATGTCGCAGTGTCATC 3'
5b, S4b, S6	Telomere southern probe	5' GGGTTACATGTTACAGGTTACA 3'
S4a	Chromosome fusion For	5' GGGTTGCAAAGTATGATTGTGGTAA 3'

Chromosome fusion Rev	5’ TGTTGAATGTCAGAACCAACTGTTGCAT 3’
Trt1 For	5’ GCAAAGAAGTTTCCTGGAATAGC 3’
Trt1 Rev	5’ GATGTAATAAAGGGTCGGCAC 3’
