# Supplementary material for: The methyl phosphate capping enzyme Bmc1/Bin3 is a stable component of the fission yeast telomerase holoenzyme
Source: Nat Commun. 2022 Mar 11;13:1277. doi: 10.1038/s41467-022-28985-3 (PMC8917221; doi:10.1038/s41467-022-28985-3)
Supplement: Supplementary file 3 — Description of Additional Supplementary Files [file 41467_2022_28985_MOESM3_ESM.pdf]

Description of Additional Supplementary Files

Supplementary data 1: Bmc1-associated RNAs detected by RIP-Seq

Transcripts with at least 1 CPM in all samples and a log2 fold change value greater than or equal to 1.

Supplementary data 2: Bmc1 PrA IP-mass spectrometry analysis of the top 50 ranked proteins

Supplementary data 3 : Distribution of Bmc1 and Pof8 in the 472 species used to construct the cladogram of Figure 6 and corresponding protein sequences.

Supplementary data 4: One way ANOVA for qPCR presented in figure 5A and S5D.
