# Supplementary material for: The methyl phosphate capping enzyme Bmc1/Bin3 is a stable component of the fission yeast telomerase holoenzyme
Source: Nat Commun. 2022 Mar 11;13:1277. doi: 10.1038/s41467-022-28985-3 (PMC8917221; doi:10.1038/s41467-022-28985-3)
Supplement: Supplementary file 8 — Reporting Summary [file 41467_2022_28985_MOESM8_ESM.pdf]

nature portfolio

Corresponding author(s): Bayfield

Last updated by author(s): Jan 6, 2022

Reporting Summary

Nature Portfolio wishes to improve the reproducibility of the work that we publish. This form provides structure for consistency and transparency in reporting. For further information on Nature Portfolio policies, see our Editorial Policies and the Editorial Policy Checklist.

Statistics

For all statistical analyses, confirm that the following items are present in the figure legend, table legend, main text, or Methods section.

n/a	Confirmed	
☐	☒	The exact sample size (n) for each experimental group/condition, given as a discrete number and unit of measurement
☐	☒	A statement on whether measurements were taken from distinct samples or whether the same sample was measured repeatedly
☐	☒	The statistical test(s) used AND whether they are one- or two-sided<br>*Only common tests should be described solely by name; describe more complex techniques in the Methods section.*
☒	☐	A description of all covariates tested
☒	☐	A description of any assumptions or corrections, such as tests of normality and adjustment for multiple comparisons
☐	☒	A full description of the statistical parameters including central tendency (e.g. means) or other basic estimates (e.g. regression coefficient) AND variation (e.g. standard deviation) or associated estimates of uncertainty (e.g. confidence intervals)
☐	☒	For null hypothesis testing, the test statistic (e.g. F, t, r) with confidence intervals, effect sizes, degrees of freedom and P value noted<br>*Give P values as exact values whenever suitable.*
☒	☐	For Bayesian analysis, information on the choice of priors and Markov chain Monte Carlo settings
☒	☐	For hierarchical and complex designs, identification of the appropriate level for tests and full reporting of outcomes
☒	☐	Estimates of effect sizes (e.g. Cohen's d, Pearson's r), indicating how they were calculated

Our web collection on statistics for biologists contains articles on many of the points above.

Software and code

Policy information about availability of computer code

Data collection	No software was used
Data analysis	Fastp(0.20.1+galaxy0), Bowtie2 (2.4.2+galaxy0), Featurecounts (2.0.1+galaxy2), EdgeR (3.34.0+galaxy2), MaxQuant (1.5.1.2)

For manuscripts utilizing custom algorithms or software that are central to the research but not yet described in published literature, software must be made available to editors and reviewers. We strongly encourage code deposition in a community repository (e.g. GitHub). See the Nature Portfolio guidelines for submitting code & software for further information.

Data

Policy information about availability of data

All manuscripts must include a data availability statement. This statement should provide the following information, where applicable:

- Accession codes, unique identifiers, or web links for publicly available datasets
- A description of any restrictions on data availability
- For clinical datasets or third party data, please ensure that the statement adheres to our policy

The mass spectrometry proteomics data have been deposited to the ProteomeXchange Consortium via the PRIDE 75 partner repository with the dataset identifier PDX023356 and 10.6019/PDX023356. RNA Seq data have been deposited in NCBI's Sequence Read Archive (SRA) database under BioProject number PRJNA776661.

Field-specific reporting

Please select the one below that is the best fit for your research. If you are not sure, read the appropriate sections before making your selection.

☒ Life sciences ☐ Behavioural & social sciences ☐ Ecological, evolutionary & environmental sciences

For a reference copy of the document with all sections, see nature.com/documents/nr-reporting-summary-flat.pdf

Life sciences study design

All studies must disclose on these points even when the disclosure is negative.

Sample size	For experiments testing for changes in abundance or expression of a protein or transcript of interest, experiments were done with biological replicates (predetermined as biological duplicate or triplicate and kept consistent throughout all like experiments) and values were compared to a control gene with statistical analysis to confirm significance (two-tailed student's t-test and, where applicable, one-way ANOVA followed by a Tukey posthoc test with a set to 0.05).
Data exclusions	No exclusions.
Replication	Most experiments performed in biological duplicate or triplicate. RIP-Seq performed in biological triplicate and mass spectrometry performed in biological duplicate. All replication attempts were successful.
Randomization	For RIP-Seq and mass spectrometry experiments all data for all genes were included in all replicates. Experimental groups were chosen as experimental (immunoprecipitation using tagged strain) versus control (immunoprecipitation of untagged strain). For northern, qPCR, and western blots, experimental groups were assigned to gene of interest versus control gene using control genes commonly used for telomerase work in S. pombe (i.e. actin for western blots and qPCR, U5 for northern blots). For telomerase assays, experimental groups were chosen as experimental (immunoprecipitation using tagged strain) and control (immunoprecipitation of untagged strain).
Blinding	For RIP-Seq and mass spectrometry analysis blinding is not relevant (whole transcriptome and proteome data analyzed). For westerns, nrotherns, and qPCR blinding is not possible (we need to know what gene we are looking for in order to know what probes or antibodies to use), but when comparing to control genes unbiased statistical tests were applied. Blinding is not relevant for telomerase assays.

Reporting for specific materials, systems and methods

We require information from authors about some types of materials, experimental systems and methods used in many studies. Here, indicate whether each material, system or method listed is relevant to your study. If you are not sure if a list item applies to your research, read the appropriate section before selecting a response.

Materials & experimental systems

n/a	Involved in the study	
☐	☒	Antibodies
☒	☐	Eukaryotic cell lines
☒	☐	Palaeontology and archaeology
☒	☐	Animals and other organisms
☒	☐	Human research participants
☒	☐	Clinical data
☒	☐	Dual use research of concern

Methods

n/a	Involved in the study	
☒	☐	ChIP-seq
☒	☐	Flow cytometry
☒	☐	MRI-based neuroimaging

Antibodies

Antibodies used	Myc (Cell Signaling, 2276S) at 1:5000, beta actin (abcam, ab8226) at 1:2500, HRP-mouse (Cell Signaling, 7076) at 1:5000, protein A (Invitrogen, PA1-26853) at 1:5000.
Validation	Antibodies were validated against untagged strains to confirm specificity for tags. HRP-mouse was validated with CST primary antibodies (Cell Signaling), myc was validated with SimpleChIP Enzymatic Chromatin IP kits), beta actin was validated by western blot in HeLa cells (abcam), protein A was validated by western blot (Invitrogen).
